# Sirolimus for Recurrent Chylothorax and Edema in an Infant with Noonan Syndrome after Resolved Hydrops Fetalis: A Case Report

**DOI:** 10.1055/a-2770-4952

**Published:** 2025-12-30

**Authors:** Mariko Hattori, Kei Tamai, Hirokazu Watanabe, Akihito Takeuchi, Makoto Nakamura, Michio Ozeki, Misao Kageyama

**Affiliations:** 1 Division of Neonatology, NHO Okayama Medical Center, Okayama, Japan; 2 Department of Pediatrics, Graduate School of Medicine, Gifu University, Gifu, Japan

**Keywords:** chylothorax, edema, Noonan syndrome, rapamycin, sirolimus

## Abstract

**Background:**

Noonan syndrome (Online Mendelian Inheritance in Man #163950) is a RASopathy caused by germline mutations in the RAS/RAF/mitogen-activated protein kinase signaling pathway and characterized by distinctive facial features, musculoskeletal abnormalities, and congenital heart defects. A subset of patients with *RIT1* mutations present with hypertrophic cardiomyopathy and generalized lymphatic anomalies. While sirolimus, a mammalian target of rapamycin inhibitor (mTOR), has been shown to suppress lymphangiogenesis, its efficacy in Noonan syndrome remains unclear.

**Case Presentation:**

We report the case of an infant with Noonan syndrome and *RIT1* mutation who developed recurrent refractory chylothorax and edema. Despite multiple interventions, including corticosteroids, octreotide, thoracic duct ligation, and pleurodesis, the patient's condition remained critical and refractory. Skin biopsy revealed lymphatic malformations. Sirolimus (0.6 mg/day) was initiated at 220 days of age, but was discontinued due to a markedly elevated serum level (88.2 ng/mL) and a lack of therapeutic effect. The patient died of *Escherichia coli* sepsis at 235 days of age.

**Conclusion:**

Although sirolimus was ineffective in this case, initiation of treatment at a lower dose may be advisable for patients with compromised hepatic function or concurrent infections. Further studies are warranted to clarify the appropriate indications, dosage, and timing of sirolimus therapy for Noonan syndrome.

## Introduction

Noonan syndrome (Online Mendelian Inheritance in Man #163950) is a RASopathy characterized by distinctive facial appearance, musculoskeletal abnormalities, and congenital heart defects. Noonan syndrome with *RIT1* mutation is associated with hypertrophic cardiomyopathy and generalized lymphatic anomalies. 1 Inoperable lymphatic malformations, which can be life-threatening, are commonly treated with interferon, corticosteroids, and propranolol. However, their efficacy is limited. 2 Sirolimus, an immunosuppressive agent, inhibits the mammalian target of rapamycin (mTOR), which regulates cell division, proliferation, and survival. 3 As an mTOR inhibitor, sirolimus suppresses lymphangiogenesis and is thought to regulate lymph production and leakage by decreasing lymphatic endothelial cell activity. 3 Herein, we report the case of an infant with Noonan syndrome who was treated with sirolimus for recurrent and refractory chylothorax and edema.

## Case Presentation

**Supplementary Video S1** Continuous, copious drainage of lymphatic fluid from the biopsy site following the release of compression.


**Fig. 1:**
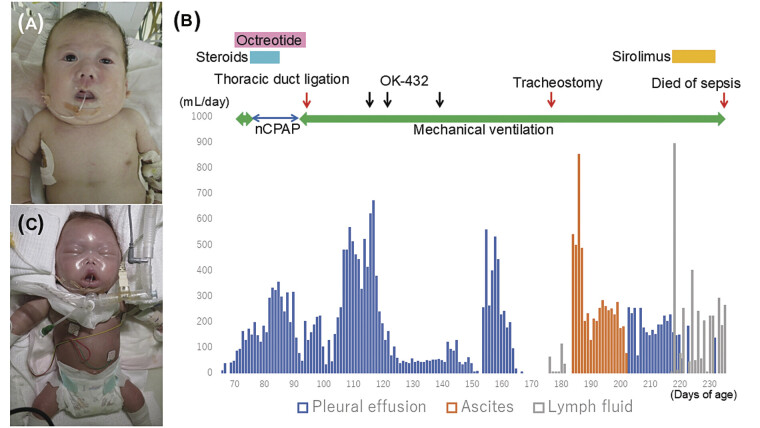
Patient's clinical features and clinical course. ( **A** ) The patient's facial appearance at 1 month of age showed low-set ears, curly hair, and a left drooping eyelid. ( **B** ) Clinical course following recurrent chylothorax. Treatments, including medium-chain triglyceride milk, octreotide, steroids, intrapleural OK-432, thoracic duct ligation, and sirolimus, were all ineffective in managing the chylothorax and subcutaneous edema. ( **C** ) Appearance at 7 months of age demonstrated severe subcutaneous edema.

The patient was the second child born to Japanese parents. The couple was not consanguineous. The patient had no family history of congenital anomalies or genetic diseases. Prenatal ultrasonography at 29 weeks of gestation revealed fetal chylothorax and subcutaneous edema. Although thoracoamniotic shunting was performed at 32 weeks of gestation, the fetal chylothorax and subcutaneous edema persisted. A male infant was delivered vaginally at 35 weeks of gestation because of worsening hydrops fetalis. The Apgar scores were 4 at 1 minute and 7 at 5 minutes. Birth weight, length, and head circumference were 2838 g, 47.5 cm, and 33.8 cm, respectively. He had the characteristic facial features of Noonan syndrome ( Fig. 1A ) and was complicated with hydrops fetalis, hypertrophic cardiomyopathy, pulmonary stenosis, atrial septal defect, and cryptorchism. He underwent chest tube placement at 0 to 3 days of age for a congenital chylothorax. Congenital chylothorax gradually improved and disappeared by 16 days of age. However, at 62 days of age, he developed a recurrent chylothorax that required thoracic drainage for respiratory distress ( Fig. 1B ). Medium-chain triglyceride milk, octreotide and steroid administration, intrapleural administration of OK-432 (a streptococcal immunotherapeutic agent commonly used in Japan for lymphatic malformations), and thoracic duct ligation were ineffective for chylothorax and subcutaneous edema ( Fig. 1C ). A skin biopsy of the inguinal region, which was suspected to be a lymphangioma on ultrasound, revealed lymphatic malformations. Significant lymphatic fluid was observed draining from the biopsy site ( Supplementary Video S1 [available in the online version only]) along with rapidly worsening subcutaneous edema and respiratory status. These findings indicate that this is a life-threatening condition that necessitates novel therapeutic interventions. Sirolimus (0.6 mg every 24 hours) was administered at 220 days of age after obtaining parental informed consent and approval from the Institutional Review Board of our hospital (#ISHOKU R1-001). However, no therapeutic effects were observed. We discontinued sirolimus administration at 232 days of age because of an extremely high trough level of sirolimus of 88.2 ng/mL (reference value: 5–15 ng/mL). At 235 days of age, the patient died of *Escherichia coli* sepsis. A known heterozygous missense mutation, c.270G > A, p.M90I, was detected in exon 4 of the *RIT1* gene, confirming the diagnosis of Noonan syndrome.

## Discussion

This report describes the case of an infant with Noonan syndrome with recurrent and refractory chylothorax and edema treated with sirolimus. The clinical course of the patient highlighted two important considerations.

This is the first reported case of sirolimus administration for recurrent and refractory chylothorax and edema in a patient with Noonan syndrome. Although previous studies have demonstrated the efficacy of sirolimus for diffuse lymphangiomatosis, 4 5 in our patient, sirolimus was not beneficial for the recurrent and refractory chylothorax and edema in Noonan syndrome. The timing of sirolimus administration may be a critical factor in the management of recurrent chylothorax and edema in patients with Noonan syndrome. Given that *RIT1* -related Noonan syndrome is caused by a gain of function in the RAS/RAF/mitogen-activated protein kinase signaling pathway, the mitogen-activated protein kinase inhibitor might be more effective than the mTOR inhibitor in such patients. 6 7

Second, careful monitoring of blood sirolimus levels is essential, particularly in life-threatening conditions. In our patient, sirolimus was administered at a standard dose (0.6 mg every 24 hours), 3 yet the blood level was markedly elevated (88.2 ng/mL). As an immunosuppressive drug, sirolimus may have contributed to the onset of sepsis in this case; however, a direct causal relationship could not be confirmed. Several factors may explain the unexpectedly high sirolimus concentration. Sirolimus is generally considered well-tolerated. This is supported by a Phase II trial using a dosing regimen of 0.8 mg/m ^2^ twice daily for complicated vascular anomalies, in which only 2 out of 61 patients discontinued treatment due to persistent adverse effects such as hematological toxicity, and no toxicity-related deaths were reported. 4 However, certain clinical conditions can predispose patients to elevated drug levels. In this case, the dose administered (0.6 mg every 24 hours) may have been slightly higher than optimal. Based on the patient's body surface area, the dose was approximately 0.2 mg per administration, given twice daily. Additionally, sirolimus is primarily metabolized in the liver. Although elevated liver enzymes and hyperbilirubinemia were not observed in this patient, the autopsy imaging revealed relative hypertrophy of the left lateral and caudate lobes of the liver, suggesting liver cirrhosis, which could have impaired drug clearance. 8 9 A pilot study has reported unexpectedly elevated sirolimus levels during concurrent infections. 10 Thus, it is plausible that the combination of an elevated sirolimus dose, hepatic dysfunction, and sepsis contributed to the abnormally high sirolimus concentration observed in the patient.

In conclusion, we reported a patient with recurrent and refractory chylothorax and edema associated with Noonan syndrome who did not respond to sirolimus treatment. Initiating therapy at a lower dose may be advisable in cases where hepatic dysfunction or concurrent infection raises concerns. Although sirolimus was ineffective in this case, its potential utility in managing the severe lymphatic complications of Noonan syndrome warrants further investigation.
